# Parallel Alterations of Functional Connectivity during Execution and Imagination after Motor Imagery Learning

**DOI:** 10.1371/journal.pone.0036052

**Published:** 2012-05-18

**Authors:** Hang Zhang, Lele Xu, Rushao Zhang, Mingqi Hui, Zhiying Long, Xiaojie Zhao, Li Yao

**Affiliations:** 1 Department of Biomedical Engineering, Peking University, Beijing, China; 2 School of Information Science and Technology, Beijing Normal University, Beijing, China; 3 State Key Laboratory of Cognitive Neuroscience and Learning, Beijing Normal University, Beijing, China; Katholieke Universiteit Leuven, Belgium

## Abstract

**Background:**

Neural substrates underlying motor learning have been widely investigated with neuroimaging technologies. Investigations have illustrated the critical regions of motor learning and further revealed parallel alterations of functional activation during imagination and execution after learning. However, little is known about the functional connectivity associated with motor learning, especially motor imagery learning, although benefits from functional connectivity analysis attract more attention to the related explorations. We explored whether motor imagery (MI) and motor execution (ME) shared parallel alterations of functional connectivity after MI learning.

**Methodology/Principal Findings:**

Graph theory analysis, which is widely used in functional connectivity exploration, was performed on the functional magnetic resonance imaging (fMRI) data of MI and ME tasks before and after 14 days of consecutive MI learning. The control group had no learning. Two measures, connectivity degree and interregional connectivity, were calculated and further assessed at a statistical level. Two interesting results were obtained: (1) The connectivity degree of the right posterior parietal lobe decreased in both MI and ME tasks after MI learning in the experimental group; (2) The parallel alterations of interregional connectivity related to the right posterior parietal lobe occurred in the supplementary motor area for both tasks.

**Conclusions/Significance:**

These computational results may provide the following insights: (1) The establishment of motor schema through MI learning may induce the significant decrease of connectivity degree in the posterior parietal lobe; (2) The decreased interregional connectivity between the supplementary motor area and the right posterior parietal lobe in post-test implicates the dissociation between motor learning and task performing. These findings and explanations further revealed the neural substrates underpinning MI learning and supported that the potential value of MI learning in motor function rehabilitation and motor skill learning deserves more attention and further investigation.

## Introduction

Motor learning, including motor execution (ME) learning and motor imagery (MI) learning has attracted increased attention among the motor function rehabilitation and motor skill learning research communities [1], [2]. Neuroimaging techniques such as functional magnetic resonance imaging (fMRI) and positron emission tomography (PET) have been used to investigate neural substrates underlying motor learning, especially motor sequence learning on MI/ME tasks [3]–[5].

Investigations revealed that executing and imagining movements possessed similar neural substrates [6], [7]. Lotze and his colleagues indicated that MI and ME shared activation in some brain areas, including the primary motor cortex (M1), supplementary motor area (SMA), premotor area (PMA), posterior parietal lobe (PPL) and cerebellar area. These areas were activated with the striatum and thalamus in MI/ME learning [4], [8], [9]. With the learning procedure, the functional similarity between MI and ME could be increased in these brain areas [10]. Moreover, it was suggested that MI/ME learning could induce parallel alteration in regional activation for both MI and ME tasks [11], [12]. Our previous study has further confirmed this finding at the functional activation level [13].

Brain areas often contribute to tasks with functional interactions between each other [14], [15]. Recently, the merits of functional connectivity analysis have encouraged more and more explorations, including current researches on the functional interactions associated with motor sequence learning [16], [17]. These studies, which mainly investigated ME learning with ME tasks, indicated that the interregional connectivity in ME tasks was attenuated after ME learning. Sun and his colleagues revealed the attenuated coupling between the SMA, PMA and M1 during executing sequential movement after ME learning. Coynel et al. reported that the functional integration among pre-SMA, PMA, PPL, and some subcortical regions decreased after ME learning. In functional connectivity analysis, the uses of correlation, coherence, beta series correlation, hierarchical integration and graph theory have all shown to be effective [16]–[20]. Among these methods, graph theory is specifically used to characterize the interactions among multiple brain regions and evaluate the information received by one particular brain region from other regions. However, potential problems may exist in interpreting results obtained by this method, especially in stimulus driven tasks. For example, the stimulus-locked response, which indicates the simultaneous response in different brain regions caused by external driven stimuli but not the intrinsic task, has been illustrated in a recent study [21], [22].

As mentioned above, studies of MI/ME learning have revealed the similarity of ME and MI tasks at the functional activation level. Furthermore, investigations have probed into the alteration of functional connectivity of ME learning with ME tasks. However, little is known about the functional connectivity associated with MI learning, especially whether a MI task shared parallel alteration of functional connectivity with a ME task after MI learning.

To address these issues, an exploratory investigation was performed at the functional connectivity level. The MI learning involving both imagination and execution tasks was examined by fMRI. We improved the graph theory method by removing the stimulus-locked response to investigate the intrinsic task-related functional connectivity in the critical areas for MI/ME tasks. According to previous researches on motor learning, we hypothesized that MI learning could induce a decrease in functional connectivity for ME/MI tasks and that the alteration might be similar with respect to ME and MI tasks. These hypotheses were tested and the results showed that parallel decreases of functional connectivity occurred in both tasks after learning.

## Methods

### Ethics Statement

The human fMRI experiment conducted in this study was approved by the Institutional Review Board of Beijing Normal University (BNU) Imaging Center for Brain Research, National Key Laboratory of Cognitive Neuroscience. All of the subjects gave written informed consent according to the guidelines set by the MRI Center of Beijing Normal University.

### Participants

Fourteen right hand-dominant subjects (seven males, mean age: 22±2 years) participated in the learning, and another twelve right hand-dominant subjects (five males, mean age 24±2 years) were recruited as control group. Participants with histories of neurological disorders, psychiatric disorders, experience with typewriters, or any experience learning to play musical instruments were excluded. All participants passed Edinburgh Handedness Inventory, Movement Imagery Questionnaire [23] and Vividness of Movement Imagery Questionnaires [24]. According to these questionnaires, we requested the participants to understand what is kinesthetic imagery, and to employ this imagery strategy during the whole experimental procedure.

### Experimental Procedure

The overall procedure of the experiment, which has been reported in our recent study [13], included familiar exercises, a pre-test, a MI learning period (experimental group)/a no-learning period (control group), and a post-test.

Outside of the scanner, all the participants were instructed that from their index to little finger, each of the four fingers of their right hand represented a single digit number: one, two, three, and four. Next, they were instructed to tap their right index finger with a metronome at 4 Hz to learn the rhythm required in the following scan session, after which they tapped 1-2-3-4 at 4 Hz for 30 s epoch. After that, they tapped the set sequence 4-2-3-1-3-4-2 at 4 Hz for 30 s epoch, and imagined tapping the set sequence at 4 Hz for 30 s epoch. These familiarization exercises were necessary for preventing confusion in each scan session and still preserved the novelty of the tasks. After finishing these exercises, the participants were prepared for pre-test in the scanner.

In pre-test, two scanning sessions, including motor execution and imagery, were completed. The two 4.5-min sessions (execution and imagery) were separated by a 5-min inter-session rest period. Each session consisted of four 30-s epochs of executing/imagining the motor sequence, interspersed with five 30-s rest blocks. The assignment of scan orders was counterbalanced across subjects. In each scanning session, a sequential finger movement task was adopted, and the press sequence was 4–2–3–1–3–4–2. Subjects attempted to execute or imagine the set sequence with the right hand at a self-paced rate of 4 Hz when PUSH was displayed on the screen, and then relaxed when REST was displayed on the screen. The participants were kept in the scanner during the whole procedure of the pre-test and the task instruction given to each participant was, “You will attend two sessions of tasks including motor execution and motor imagery. The type of the task will be displayed on the screen before the task starting. If the task is motor execution, you need to tap 4-2-3-1-3-4-2 with your right hand fingers as fast as the rate which you have just learned outside the scanner, and if the task is motor imagery, you need to imagine tapping 4-2-3-1-3-4-2 with your right hand fingers as fast as the pace which you have just learned outside the scanner.” The descriptions of the task type, which were displayed to the subject via a mirror mounted on the head coil, were presented visually on a semi-transparent screen at the end of the scanner bore. Cushions inside the head coil were used to reduce head movement. The sequence tapping was performed with a four-button response pad, and the response pad was connected to a computer running the E-prime program (Psychology Software Tools, PA, USA) to record the responses. After test, participants should provide qualitative description of performing in order to controlling the imagery. The contents of the qualitative description was patterned from Movement Imagery Questionnaire [23] and included seven rating levels (1, Very Hard to feel; 2, Hard to feel; 3, Somewhat hard to feel; 4, Neutral (not easy not hard); 5, Somewhat easy to feel; 6, Easy to feel; 7, Very easy to feel). Each Participant should rate the levels reliably, and no participants rated the level lower than 5.

During the learning period, 14 motor imagery practice sessions were performed over 14 consecutive days to guarantee sufficient learning. In the control group, participants did not attend any learning during the 14 days. In the experimental group, participants were trained under the inspection of the experimenter, and their right hand was covered by a cardboard box to prevent visual feedback. Participants also should provide the similar qualitative description of performing after finishing every learning session as pre-test. During the whole learning periods, no participants rated the level lower than 5. We further calculated the mean rating for each participant over 14 days and then, checked the mean rating as well as the standard deviation over 14 participants, the results (14 participants, Mean rating: 5.9±0.7) ensured that the participants performed the motor imagery learning adequately. The following instruction was provided to the participants in each learning session, “You will attend the motor imagery learning. The learning includes two sections, metronome-pacing, and self-pacing. The metronome-pacing will last for 15 min, and you should imagine tapping 4-2-3-1-3-4-2 with your right hand fingers repeatedly as fast as the pace of the metronome. Then, you will attend the self-pacing section, and imagine tapping 4-2-3-1-3-4-2 with your right hand fingers repeatedly as fast as the pace controlled by yourself. This section will also last for 15 min.” Each learning session consisted of two sections. One section, lasting for 15 min, was paced by the metronome, and the other, also lasting for 15 min, was paced by participants themselves. Each section consisted of repetitive cycles of rest (30 s), and imagery practice (30 s). At the first two practice days, participants were paced at 2 Hz according to the behavioral results of the pre-test. This requirement was found to be important in a previous study, and helped to ensure that participants could focus on establishing a representation of the sequence order [10]. From the third day onward, the frequency of pacing was increased to 4 Hz to encourage participants to improve the tapping rate. Following the last learning session, all of the participants were tested again in the scanner. The requirement for the tapping rate was also 4 Hz, and the procedure and instructions of post-test were identical to the pre-test.

### fMRI Data Acquisition

Brain scans were performed at the MRI Center of Beijing Normal University using a 3.0-T Siemens whole-body MRI scanner. A single-shot T2*-weighted gradient-echo, EPI sequence was used for functional imaging acquisition, with the parameters: TR/TE/flip angle = 3000 ms/40 ms/90°, acquisition matrix = 64×64; field of view (FOV) = 240 mm; and slice thickness = 5 mm with no inter-slice gap. Thirty-two axial slices parallel to the AC-PC line were obtained in an interleaved order to cover the entire cerebrum and cerebellum.

### Data Processing

The study was performed based on the processed data of our previous research. The functional images were first realigned, spatially normalized into standard stereotaxic space (EPI template provided by the Montreal Neurologic Institute, MNI), re-sliced to 3×3×4 mm voxels, and smoothed with an 8×8×8 full-width at half maximum (FWHM) Gaussian kernel using SPM8 software (Statistical Parametric Mapping; http://www.fil.ion.ucl.ac.uk/spm). The first five images in each series were removed from further analysis. Using rest as the baseline, general linear model (GLM) analysis was applied to each subject’s data processed by a high-frequency filter and global scaling with SPM8. Then, task-related t-contrast images were calculated using the t-statistic for each subject.

A two-way within-subjects ANOVA test, treating subjects as a random factor, was performed respectively within experimental group and control group. The ANOVA model of experiment group used learning (pre-test and post-test; between-subjects; and fixed effect), task (motor execution and motor imagery; within-subjects; and fixed effect) as the main factors to assess regional activities for ME/MI task in the pre- and post-test sessions. An identical ANOVA model was employed for the control group to examine regional activities for ME/MI task in the pre- and post-test after the no-learning period.

### Selection of Regions of Interest (ROIs)

Considering the structural and functional alignments, we defined regions of interest (ROIs) according to results of group-level and individual-level analysis. The SMA, M1, PMA, cerebellum, striatum, PPL, and thalamus are suggested as the critical regions in motor sequence learning [4], [9], [13]. Therefore we paid close attention to these regions in this study. However, the recruiting of M1 is still controversial in MI tasks, and we did not find any activities in the right M1 at the reduced threshold of p<0.05 for MI tasks. Therefore, we finally focused on 13 ROIs for the ME task and 12 ROIs for the MI task (excluding the right M1). The ROIs were defined according to the procedures of previous studies [20], [25]. We first defined 10-mm-radius spheres around the maxima of the focused brain regions based on the group’s t-contrast maps. The coordinates of these group ROIs are shown in (see details in Tables S1, S2, S3, S4). Thereafter, individual ROIs were further defined within these group ROIs as follows. Taking a given group ROI as a mask, the voxel with the maximum t-value within this mask was picked up as the individual peak voxel. Then a 6-mm-radius sphere around this peak voxel was taken as an individual ROI. After that, for each subject, the averaged time series was extracted from each individual ROI of both pre and post MI/ME tasks in the experimental and control groups for further functional connectivity analyses.

### Functional Connectivity Analyses

In this study, the graph theory method, which possesses advantages in describing the functional connectivity of multiple brain regions, was used to examine the MI/ME task before and after MI learning [20].

For the graph theory method, the ROIs are denoted by nodes in a graph, and the links between the nodes indicate the functional interaction between them. The interregional connectivity between the node *i* and the node *j* is defined as



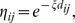
where 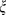 is a real positive constant measuring how the strength of the functional interaction decreases with the distance between the two nodes, and we set it equal to 2 for this study as the previous studies [20], [26]. 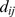 represents the distance between the two nodes, calculated as follows:



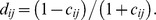
Considering the influence of a stimulus-locked response in the task state, 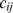 in our study represents the partial correlation coefficient of two averaged time series,



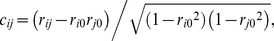
where 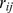 denotes the Pearson correlation coefficient between the two time series of node *i* and node *j*. 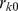 is the Pearson correlation coefficient between the time series of node *k* and the reference function which is modeled by the stimulus presentation paradigm in our study.

To measure the connectivity degree 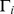 of a node *i* in a graph, we define the sum of all the interregional connectivity between i and all other nodes as 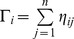. It illustrates the total functional interaction information that node *i* receives from other nodes. Thus, the node with larger 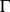 is more functionally connected to other nodes.


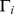
 is further normalized as 
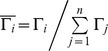
. Two-way ANOVA repeated measures were carried out for examining the 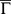 of each ROI in MI/ME tasks. The ANOVA model for each ROI used learning (pre-test and post-test; within-subjects) and group (experimental group and control group; between-subjects) as the main factors. First, the 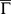 of ROIs showing an interaction effect between learning and group were detected. Then, differences between the pre- and post-tests for each group and differences between the pre-tests for the two groups were further examined. These differences were Bonferroni-corrected within the analysis model for each ROI. 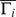 is a measurement of the connectivity degree of node i among multiple nodes; namely, it measures the total interregional connectivity between node i and all the other nodes. Moreover, it is necessary to further investigate the specific link related to node i in the alteration of 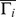. Thus, as to the nodes which were significantly altered in 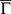, further exploration on the interregional connectivity between two nodes was carried out. Specifically, for each node *i*, the interregional connectivity 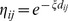 for each 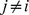 was analyzed statistically by paired t-test between pre- and post- MI/ME tasks and further corrected with Bonferroni method.

**Figure 1:**
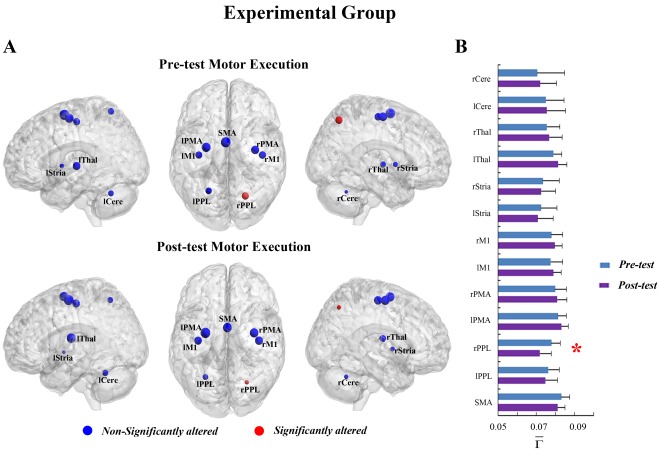
The connectivity degree 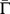 of pre-tests and post-tests for all ROIs in the motor execution task of the experimental group. (A) The surface visualization of all 13 ROIs with node sizes indicating their relative value of 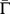. Red indicates that 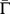 of the ROIs were significantly altered after motor imagery learning, while blue indicates that 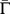 of the ROIs were not significantly altered after motor imagery learning. (B) 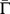 of pre-tests and post-tests for all ROIs (* represents the significant alterations, corrected p<0.05).

### Behavior and Behavior-Connectivity Analyses

Completed button pressing was electronically recorded for the four 30-s epochs of the execution task inside the MRI scanner during the pre- and post-test scanning session. The mean execution rate and errors were calculated for each test. Differences in the mean execution rate and number of errors between pre-test and post-test conditions have been analyzed for both the experimental and control groups using a paired t-test.

The relationship between the improvement in motor behavior and the changes in functional connectivity was further investigated in the execution task of the experimental group. The connectivity degree (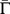) and the interregional connectivity (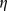) which were significantly altered after learning were involved in the following analysis. Based on the behavioral and functional connectivity results, the linear regression approach was employed to evaluate the correlations between the connectivity degree or interregional connectivity and the tapping rate of the execution task in the pre- and post-tests, respectively.

**Figure 2:**
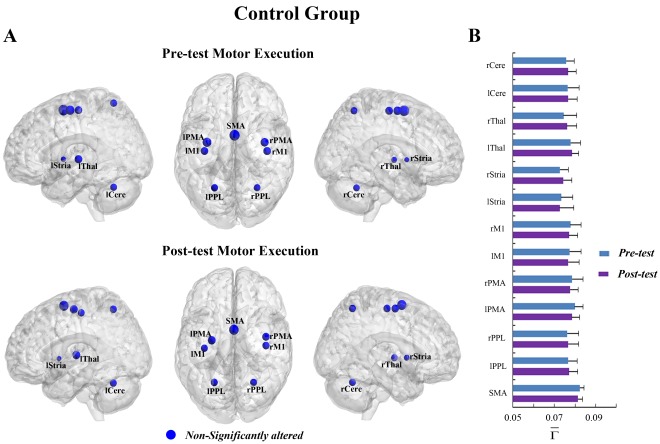
The connectivity degree 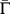 of pre-tests and post-tests for all ROIs in the motor execution task of the control group. (A) The surface visualization of all 13 ROIs with node sizes indicating their relative value of 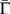. Blue indicates that 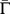 of the ROIs were not significantly altered after motor imagery learning. (B) 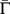 of pre-tests and post-tests for all ROIs.

## Results

### Alterations of Connectivity Degree

In the ME task, a significant interaction effect between learning and group was found in the right PPL (rPPL) (F = 6.480, p<0.05, see Table S5). Figure 1A shows that MI learning has altered the connectivity degree of the rPPL for the experimental group but not for the control group (Figure 2A and 2B). In the experimental group, a significant decrease in 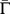 was detected in the rPPL (F = 12.247, corrected p<0.005, Figure 1B). Such an alteration could be observed in each subject, indicating a consistency of alteration existed across all subjects (see Figure S1A). Furthermore, there was no difference between the experimental and control groups for the rPPL at pre-test (baseline condition) (F = 0.702, p>0.05). Other ROIs also showed trends toward alteration after learning for the experimental group, though they were not significant. The trends toward increases in 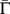 occurred in ROIs of the bilateral PMA (rPMA and lPMA), bilateral M1 (rM1 and lM1), bilateral thalamus (rThal and lThal) and bilateral cerebellum (rCere and lCere), and the trends toward decreases in 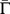 were detected in the SMA, bilateral PPL (rPPL and lPPL) and bilateral striatum (rStria and lStria) (Figure 1B).

**Figure 3:**
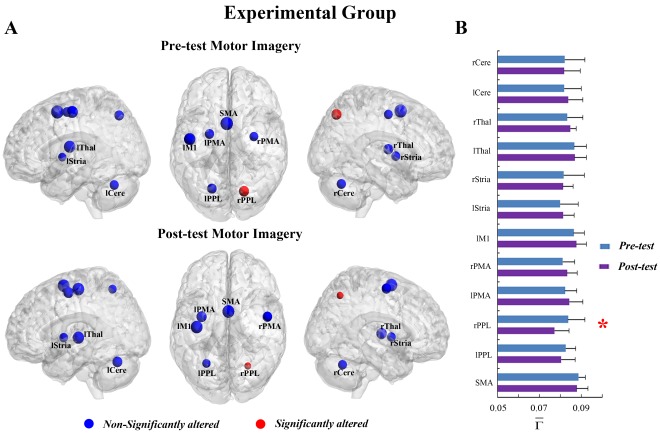
The connectivity degree 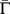 of pre-tests and post-tests for all ROIs in the motor imagery task of the experimental group. (A) The surface visualization of all 12 ROIs with node sizes indicating their relative value of 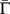. Red indicates that 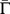 of the ROIs were significantly altered after motor imagery learning, while blue indicates that 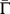 of the ROIs were not significantly altered after motor imagery learning. (B) 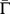 of pre-tests and post-tests for all ROIs (* represents the significant alterations, corrected p<0.05).

**Figure 4:**
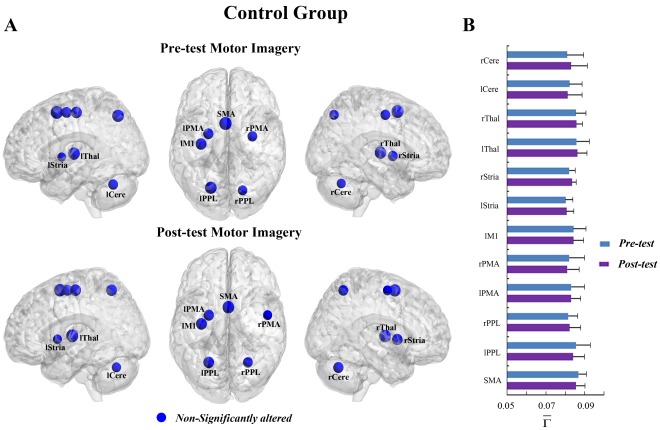
The connectivity degree 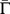 of pre-tests and post-tests for all ROIs in the motor imagery task of the control group. (A) The surface visualization of all 12 ROIs with node sizes indicating their relative value of 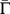. Blue indicates that 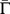 of the ROIs were not significantly altered after motor imagery learning. (B) 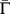 of pre-tests and post-tests for all ROIs.

In the MI task, a significant interaction effect between learning and group was also detected in the rPPL (F = 5.574, p<0.05, see Table S5). Figure 3A shows that the connectivity degree of the rPPL was altered by learning for the experimental group but not for the control group (Figure 4A and 4B). A significant decrease in 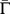 was observed in the rPPL (F = 10.076, corrected p<0.005, Figure 3B). Such alteration occurred in most subjects (13/14) (See Figure S1B). No difference between the two groups was found for the rPPL at pre-test (baseline condition) (F = 0.851, p>0.05). The trends toward alteration were also found in other ROIs, though they were not significant. 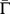 of several ROIs including the bilateral PMA, left M1, left striatum, bilateral thalamus and left cerebellum showed trends toward increases after MI learning, whereas 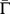 of the other ROIs including the SMA, bilateral PPL, right striatum and right cerebellum showed trends toward decreases after MI learning (Figure 3B).

**Figure 5:**
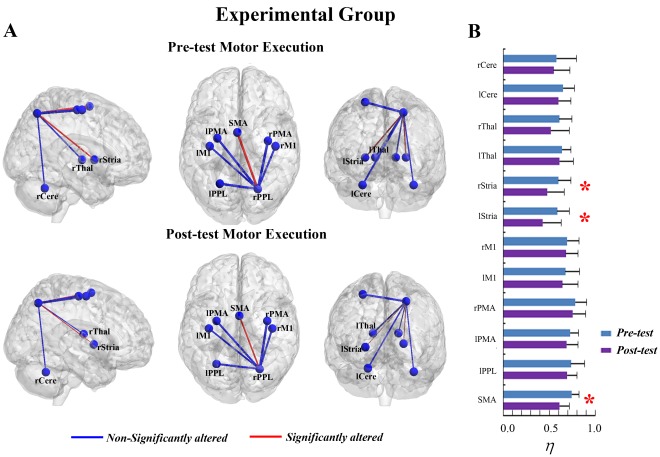
The interregional connectivity 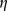 between the rPPL and other ROIs of pre-tests and post-tests in the motor execution task of the experimental group. (A) The surface visualization of all 13 ROIs with line width indicating the relative value of 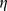. Red indicates the 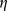 were significantly altered after motor imagery learning, while blue indicates the 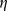 were not significantly altered after motor imagery learning. (B) The 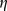 of pre-tests and post-tests between the rPPL and other ROIs (* represents the significant alterations, corrected p<0.05).

**Figure 6:**
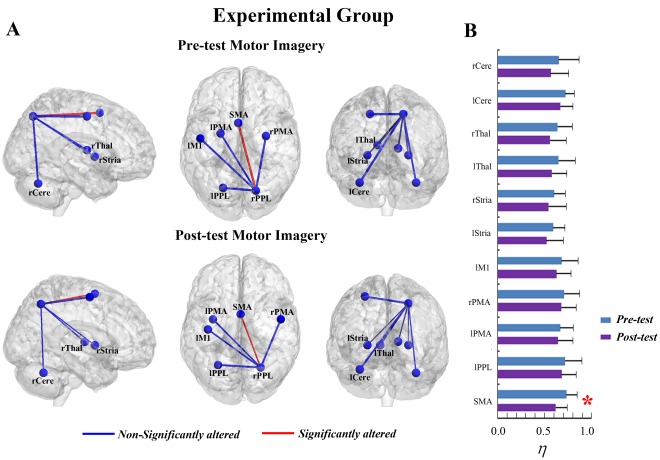
The interregional connectivity 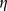 between the rPPL and other ROIs of pre-tests and post-tests in the motor imagery task of the experimental group. (A) The surface visualization of all 12 ROIs with line width indicating the relative value of 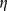. Red indicates the 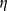 were significantly altered after motor imagery learning, while blue indicates the 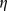 were not significantly altered after motor imagery learning. (B) The 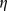 of pre-tests and post-tests between the rPPL and other ROIs (* represents the significant alterations, corrected p<0.05).

**Figure 7:**
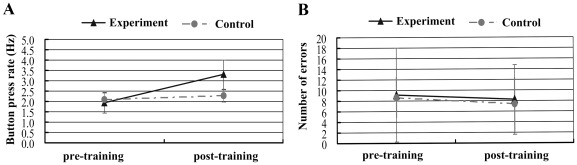
Mean button press rate (A) and mean number of errors (B) for pre-tests and post-tests of the experimental group and the control group.

**Table 1:** The correlation between the tapping rate and 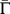 or 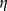 for the experimental group.

Condition	pre-/post-test	r	F(1, 12)	p
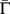				
rPPL	pre-test	0.077	0.071	0.794
rPPL	post-test	0.058	0.041	0.843
*η*				
rPPL-SMA	pre-test	0.327	1.434	0.254
rPPL-SMA	post-test	−0.190	0.450	0.515
rPPL-rStria	pre-test	0.102	0.127	0.728
rPPL-rStria	post-test	0.031	0.011	0.917
rPPL-lStria	pre-test	−0.069	0.058	0.814
rPPL-lStria	post-test	0.035	0.015	0.906

Note. Abbreviations: rPPL–right posterior parietal lobe; SMA–supplementary motor area; rStria–right Striatum; lStria–left Striatum.

### Alterations of Interregional Connectivity

The interregional connectivity (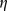) between the rPPL and other ROIs was measured in further analysis. For the ME task of the experimental group, the 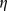 between the rPPL and SMA as well as the 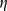 between the rPPL and bilateral striatum were attenuated after MI learning at a significant level (Figure 5A). The decrease in the 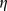 between the rPPL and SMA was significant (Figure 5B; T (13) = 6.611, corrected p<0.001) and consistent across all subjects at the individual level (see details in Figure S2A). The 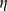 between the rPPL and the left/right striatum significantly decreased after learning (Figure 5B; left striatum: T (13) = 5.263, corrected p<0.005; right striatum: T (13) = 4.672, corrected p<0.01). Such alterations were also consistent across all subjects (see details in Figure S2A). As for the MI task, the 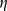 between the rPPL and most other ROIs were moderately altered after MI learning except the 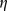 between the rPPL and SMA (Figure 6A). The decrease in the 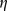 between the rPPL and SMA was significant after MI learning (Figure 6B; T (13) = 5.895, corrected p<0.001). Such alteration was consistent across all subjects at the individual level (see details in Figure S2B).

### Behavior Results

In the experimental group, the participants performed the sequence tapping at the mean execution rate of 2.0 Hz in pre-test scanning (Figure 7A). The solid line in Figure 7A illustrates that the mean execution rate of the experimental group was significantly faster in the post-test than the pre-test (T (13) = 9.27, p<0.001), however the rate did not reach the required rate of 4 Hz. In addition, the difference in the number of errors between the pre- and post-test sessions was not significant (T (13) = 0.42, p>0.05; solid line in Figure 7B).

In the control group, the dashed line in Figure 7A indicates that the mean execution rate of the participants moderately increased from 2.1 Hz, seen in the pre-test phase to 2.3 Hz in the post-test (T (11) = 3.35, p<0.05). As to the execution rate, the extent of the changes from pre-test to post-test for the control group was 0.2 Hz, which is significantly less than the result of 1.4 Hz for the experimental group (T = 7.79, p<0.001). The number of errors observed during the post-test vs. the pre-test was also not significant for the experimental group (T (11) = 0.76, p>0.05; dashed line in Figure 7B).

In the experimental group, there is no significant result to indicate that the alterations in connectivity degree (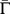) or interregional connectivity (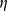) were associated with the improvement in tapping rate. The connectivity degree of the rPPL was not correlated with the tapping rate of the ME task in the pre-/post-tests, although the significant alteration of connectivity degree was found in the rPPL for both tasks after learning (see details in Table 1). Similarly, the interregional connectivity between the rPPL and SMA or striatum was significantly decreased after learning in ME tasks for the experimental group, but the interregional connectivity did not have correlations with the tapping rate in the pre-/post-tests (for detailed results, see Table 1).

## Discussion

Using graph theory, the present study investigated the functional connectivity of MI learning on both MI and ME tasks. After MI learning, parallel alterations were detected in both MI and ME tasks. Importantly, such alterations were specific to the experimental group but not to the control group, indicating that such alterations of functional connectivity were induced by MI learning, not by other effects. MI and ME tasks showed similar significant decreases of connectivity degree in the rPPL after learning. Further exploration on interregional connectivity between the rPPL and other ROIs revealed that significant alterations induced by MI learning occurred in the SMA for both tasks.

### Parallel Alterations of Connectivity Degree

The investigation of connectivity degree was performed among the ROIs including the M1, PMA, PPL, SMA, cerebellum, thalamus, and striatum. These areas have been suggested to be critical regions in executing and imagining sequential movement [4], [8], [9]. Although these ROIs were defined according to the maximal t-value voxel in the analysis, the anatomical specificity of the motor cortical areas was also taken into our considerations. The role and activation of M1 were still controversial in MI tasks [27], [28]. In our study, the left M1 was activated during motor imagery in the pre- and post-tests of the experimental/control groups, and no activity was detected in the right M1 during the whole process. One possible explanation for this issue was that the left motor cortex initiated a movement regardless of which hand was used [29], thus, the differences in functional activities between the left M1 and right M1 may result from the laterality effect of motor imagery. Accordingly, the right M1 was not involved in the analysis on the MI task. Many studies have indicated that PMA was involved two sections, dorsal and ventral areas [30], [31]. The two areas were recruited in different circuits as described by tracer studies in primates. Dorsal PMA mainly receives inputs from superior parietal lobe, while ventral PMA receives inputs form inferior lobe [32], [33]. The PPL, in our study, was specifically in superior parietal lobe, and then the ROI of PMA was exactly located in dorsal PMA for all conditions. The SMA was anatomically and functionally divided into pre and proper parts by some studies [34]–[36]. However, such conclusions were mainly proposed from the experiments of actual execution, and the SMA has been suggested to possess functional distinctions between motor execution and imagery [35]. In our study, the pre-SMA and SMA-proper were not activated independently in execution/imagery tasks of the experimental or control groups, and the peak activation voxels were mainly on the anatomical edge between the two parts of the SMA in all conditions. Therefore, we did not assume the functional distinction in the pre-SMA and SMA-proper during investigating MI learning, and the two parts of the SMA were considered as a whole ROI in the relevant analysis.

In the experimental group, MI learning induced a decrease in functional connectivity during execution/imagination. Such results were consistent with the findings of previous studies on ME learning [16], [17]. In our study, the ME and MI tasks shared parallel significant decreases of connectivity degree in the rPPL after MI learning. Specifically, the effects were found in the right superior parietal lobe (Brodmann area 7) for both tasks. This area is generally considered to be a part of the motor system, which was suggested to be associated with many functions in cognitive tasks, such as visuomotor transformations, attention, sensory-motor integration and spatial coding [37]–[41]. The experiment in the current study mainly involved internal guided learning with motor imagery, and the ROIs we focused were mainly associated with motor functions. Thus, sensory-motor integration and spatial coding may be possible explanations for the effects in the PPL for both tasks.

In general, the motor schema was established with motor learning process from novelty to automaticity [42], [43]. At the novelty phase, sensory-motor information was processed by several regions, such as the SMA, PMA, M1 and striatum [44], [45]. Such information could be further integrated in the PPL to generate internal movement images and encode the spatial locations of movement as motor schema [46]–[49]. The movements were gradually automated with this process. After MI learning, the motor schema was established and then the rPPL may play a role in storing and retrieving motor schema [50], [51]. Thus, the decrease in connectivity degree of the rPPL was probably due to the established motor schema [50]. Although trends towards decrease of connectivity degree were also observed in the lPPL, such alterations did not reach a significant level. These results were potentially related to the experiment design, which requested the participants to execute/imagine a finger tapping task with their right hands. It was interesting to note that the significant decrease of connectivity degree in the PPL induced by MI learning was parallel in the MI and ME tasks, which also supported the importance of the PPL especially the superior parietal lobe in MI learning [52]. Such effects in the PPL for both tasks potentially indicated that the motor schema could be established without motor output and may be not specific to mental imagination or real execution. However, these interpretations still require targeted investigations. Moreover, the connectivity degree is a measurement of the changes relative to the total connectivity of all the ROIs, which ignores the interregional connectivity between ROIs. The decreases in connectivity degree implicate the potential functional dissociation between the rPPL and other ROIs for both the MI and ME tasks after learning. Thus, we further assessed the interregional connectivity between the rPPL and other ROIs in this study to clarify this issue.

### Parallel Alterations of Interregional Connectivity

A parallel decrease of interregional connectivity between the rPPL and SMA was detected in both MI and ME tasks after MI learning. The SMA, as a crucial region in the motor cortex, was implicated in establishing the motor schema during motor learning [4], [53]. Strong relationships between the PPL (specifically superior parietal lobe) and SMA have been identified in MI tasks [54]. Thus, the interregional connectivity between the SMA and PPL may be crucial in learning during the novelty phase. After learning, the tasks were performed automatically with the established motor schema, and then the rPPL may play a role in the maintenance and retrieving of the motor schema, while the SMA may be more associated with motor control [34], [50], [51]. The significantly decreased interregional connectivity between the rPPL and SMA indicated attenuated functional interaction between the two regions. Therefore, these results further implicated the potential dissociation between motor learning and task performing. In the ME task, significant decreases were also detected in interregional connectivity between the rPPL and bilateral striatum after MI learning. Studies employing ME tasks have indicated that the striatum received sensory-motor information and may be further involved in the establishment of motor schema for ME learning [45], [55], [56]. These decreases observed in the ME task therefore suggested that the establishment of motor schema was more related to the striatum in ME tasks than in MI tasks. Moreover, the ROIs of striatum in our study were exactly located in the putamen for all conditions. The putamen was associated with motor functions, such as behavior control in motor tasks [57], [58]. Thus, these decreases in interregional connectivity between the rPPL and putamen for ME tasks in the post-test also implied the dissociation between motor learning and task performing. This finding combining with the decreases of interregional connectivity in rPPL-SMA during execution tasks after learning then supported the view that the SMA may mediate the execution of learned sequential movements with the putamen after learning [59]. Furthermore, recent studies have revealed that the PPL and SMA are recruited in different functional circuits of motor function. The SMA is a key node in the frontal-motor circuit, and the PPL is a critical region in the parietal-premotor circuit which was suggested to contain the learned contents [60]–[62]. Then, the decreased interregional connectivity potentially indicated an attenuated functional interaction between the parietal-premotor circuit and the frontal-motor circuit after MI learning. Such issues will be further examined in our future studies.

### Behavior-Connectivity

In the experimental group, significant behavioral improvements in the tapping rate and a slight decrease in the number of errors were observed during execution tasks after learning, however, there were not any significant relationships between tapping rate and functional connectivity (including connectivity degree and interregional connectivity). There were two potential explanations for such results. One was the limited number of participants, and another was that functional connectivity may not be associated with motor behavior directly. Interestingly, our previous study showed that tapping rate was significantly correlated with the activity (beta-value) of the right PMA, whether in the pre-test or post-test [13]. Thus, motor behavior was probably related to the activities of specific brain regions, and the changes in connectivity were more likely associated with senior processes of motor learning, e.g. establishing motor schema.

### Summary and Limitations

We summarize by suggesting that MI learning could induce parallel alterations of functional connectivity during executing and imaging. In this process, the attenuated connectivity degree of the rPPL implicates the prominent role of the PPL in establishing the motor schema. The decreased interregional connectivity between the rPPL and SMA potentially suggested the dissociation between motor learning and task performing in post-tests. These alterations at the functional connectivity level were parallel in both tasks, implicating the value of MI learning in motor function rehabilitation as well as motor skill learning. However, there exist several limitations in the current study. Our research, as an exploratory investigation, was more focused on the intrinsic task-related connectivity for motor execution/imagery before/after MI learning. Thus, graph theory was improved by removing the stimulus-locked response according to the previous study [21]. These removed responses was correlated with the stimulus presentation paradigm, and therefore some worthy results in functional connectivity might be missed by doing so, if the task-related functional connectivity possesses computational correlation with the stimulus presentation paradigm. Moreover, increasing the number of participants and focusing on specific ROIs may provide further convincing results besides the present study. In any case, MI learning, as an important part of motor learning, is worthy of further investigations at different levels.

## Supporting Information

Figure S1

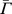
 **of the rPPL for each subject of the experimental group in motor execution/imagery task, (A) motor execution task, (B) motor imagery task.**
(TIF)

Figure S2

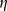
 **showing significant alteration between the rPPL and some other ROIs for each subject of the experimental group in motor execution/imagery tasks, (A) 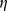 between the rPPL and SMA/left striatum/right striatum for each subject, motor execution task, (B) 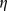 between the rPPL and SMA for each subject, motor imagery task.**
(TIF)

Table S1 **The coordinates and t-value of the peak voxel within group ROIs for motor execution and motor imagery tasks at pre-test for the experimental group.**
(DOC)

Table S2 **The coordinates and t-value of the peak voxel within group ROIs for motor execution and motor imagery tasks at post-test for the experimental group.**
(DOC)

Table S3 **The coordinates and t-value of the peak voxel within group ROIs for motor execution and motor imagery tasks at pre-test for the control group.**
(DOC)

Table S4 **The coordinates and t-value of the peak voxel within group ROIs for motor execution and motor imagery tasks at post-test for the control group.**
(DOC)

Table S5 **The interaction effect between group and learning for each ROI in motor execution and motor imagery tasks.**
(DOC)

## References

[1] Lotze M, Halsband U (2006). Motor imagery.. J Physiol-Paris.

[2] Sharma N, Pomeroy VM, Baron JC (2006). Motor Imagery: A Backdoor to the Motor System After Stroke?. Stroke.

[3] Peigneux P, Laureys S, Fuchs S, Destrebecqz A, Collette F (2003). Learned material content and acquisition level modulate cerebral reactivation during posttraining rapid-eye-movements sleep.. Neuroimage.

[4] Hikosaka O, Nakamura K, Sakai K, Nakahara H (2002). Central mechanisms of motor skill learning.. Curr Opin Neurobiol.

[5] Argyelan M, Carbon M, Ghilardi MF, Feigin A, Mattis P (2008). Dopaminergic suppression of brain deactivation responses during sequence learning.. The Journal of Neuroscience.

[6] Hanakawa T, Immisch I, Toma K, Dimyan MA, Van Gelderen P (2003). Functional properties of brain areas associated with motor execution and imagery.. J Neurophysiol.

[7] Lotze M, Montoya P, Erb M, Hülsmann E, Flor H (1999). Activation of cortical and cerebellar motor areas during executed and imagined hand movements: an fMRI study.. J Cognitive Neurosci.

[8] Guillot A, Collet C, Nguyen VA, Malouin F, Richards C (2008). Functional neuroanatomical networks associated with expertise in motor imagery.. Neuroimage.

[9] Ungerleider LG, Doyon J, Karni A (2002). Imaging Brain Plasticity during Motor Skill Learning.. Neurobiol Learn Mem.

[10] Lacourse MG, Orr ELR, Cramer SC, Cohen MJ (2005). Brain activation during execution and motor imagery of novel and skilled sequential hand movements.. Neuroimage.

[11] Lafleur MF, Jackson PL, Malouin F, Richards CL, Evans AC (2002). Motor learning produces parallel dynamic functional changes during the execution and imagination of sequential foot movements.. Neuroimage.

[12] Jackson PL, Lafleur MF, Malouin F, Richards CL, Doyon J (2003). Functional cerebral reorganization following motor sequence learning through mental practice with motor imagery.. Neuroimage.

[13] Zhang H, Xu L, Wang S, Xie B, Guo J (2011). Behavioral improvements and brain functional alterations by motor imagery training.. Brain Res.

[14] Kelly AM, Garavan H (2005). Human functional neuroimaging of brain changes associated with practice.. Cereb Cortex.

[15] Büchel C, Friston K (2000). Assessing interactions among neuronal systems using functional neuroimaging.. Neural Networks.

[16] Sun FT, Miller LM, Rao AA, D’Esposito M (2007). Functional connectivity of cortical networks involved in bimanual motor sequence learning.. Cereb Cortex.

[17] Coynel D, Marrelec G, Perlbarg V, Pélégrini-Issac M, Van de Moortele PF (2010). Dynamics of motor-related functional integration during motor sequence learning.. Neuroimage.

[18] Uddin LQ, Clare Kelly AM, Biswal BB, Xavier Castellanos F, Milham MP (2009). Functional connectivity of default mode network components: correlation, anticorrelation, and causality.. Hum Brain Mapp.

[19] Rissman J, Gazzaley A, D’Esposito M (2004). Measuring functional connectivity during distinct stages of a cognitive task.. Neuroimage.

[20] Jiang T, He Y, Zang Y, Weng X (2004). Modulation of functional connectivity during the resting state and the motor task.. Hum Brain Mapp.

[21] Sun FT, Miller LM, D’Esposito M (2004). Measuring interregional functional connectivity using coherence and partial coherence analyses of fMRI data.. Neuroimage.

[22] Arfanakis K, Cordes D, Haughton VM, Moritz CH, Quigley MA (2000). Combining independent component analysis and correlation analysis to probe interregional connectivity in fMRI task activation datasets.. Magn Reson Imaging.

[23] Hall CR, Martin KA (1997). Measuring movement imagery abilities: A revision of the Movement Imagery Questionnaire.. Journal of Mental Imagery.

[24] Isaac A, Marks DF, Russell DG (1986). An instrument for assessing imagery of movement: The Vividness of Movement Imagery Questionnaire (VMIQ).. Journal of Mental Imagery.

[25] Hattori N, Shibasaki H, Wheaton L, Wu T, Matsuhashi M (2009). Discrete parieto-frontal functional connectivity related to grasping.. J Neurophysiol.

[26] López L, Sanjuán MAF (2002). Relation between structure and size in social networks.. Phys Rev E.

[27] Dechent P, Merboldt KD, Frahm J (2004). Is the human primary motor cortex involved in motor imagery?. Cognitive Brain Res.

[28] Alkadhi H, Brugger P, Boendermaker SH, Crelier G, Curt A (2005). What disconnection tells about motor imagery: evidence from paraplegic patients.. Cereb Cortex.

[29] Johansson RS, Theorin A, Westling G, Andersson M, Ohki Y (2006). How a lateralized brain supports symmetrical bimanual tasks.. PLoS biology.

[30] Kantak SS, Stinear JW, Buch ER, Cohen LG (2011). Rewiring the Brain: Potential Role of the Premotor Cortex in Motor Control, Learning, and Recovery of Function Following Brain Injury.. Neurorehabilitation and Neural Repair.

[31] Hoshi E, Tanji J (2006). Differential involvement of neurons in the dorsal and ventral premotor cortex during processing of visual signals for action planning.. J Neurophysiol.

[32] Matelli M, Govoni P, Galletti C, Kutz DF, Luppino G (1998). Superior area 6 afferents from the superior parietal lobule in the macaque monkey.. The Journal of comparative neurology.

[33] Rozzi S, Calzavara R, Belmalih A, Borra E, Gregoriou GG (2006). Cortical connections of the inferior parietal cortical convexity of the macaque monkey.. Cereb Cortex.

[34] Kasess CH, Windischberger C, Cunnington R, Lanzenberger R, Pezawas L (2008). The suppressive influence of SMA on M1 in motor imagery revealed by fMRI and dynamic causal modeling.. Neuroimage.

[35] Stephan KM, Fink GR, Passingham RE, Silbersweig D, Ceballos-Baumann AO (1995). Functional anatomy of the mental representation of upper extremity movements in healthy subjects.. J Neurophysiol.

[36] Sakai K, Hikosaka O, Miyauchi S, Takino R, Sasaki Y (1998). Transition of brain activation from frontal to parietal areas in visuomotor sequence learning.. The Journal of Neuroscience.

[37] Behrmann M, Geng JJ, Shomstein S (2004). Parietal cortex and attention.. Curr Opin Neurobiol.

[38] Chafee MV, Averbeck BB, Crowe DA (2007). Representing spatial relationships in posterior parietal cortex: single neurons code object-referenced position.. Cereb Cortex.

[39] Fogassi L, Luppino G (2005). Motor functions of the parietal lobe.. Curr Opin Neurobiol.

[40] Wolperts DM, Goodbody SJ, Husain M (1998). Maintaining internal representations: the role of the human superior parietal lobe.. Nat Neurosci.

[41] Wenderoth N, Toni I, Bedeleem S, Debaere F, Swinnen SP (2006). Information processing in human parieto-frontal circuits during goal-directed bimanual movements.. Neuroimage.

[42] Schmidt RA (2003). Motor schema theory after 27 years: reflections and implications for a new theory.. Res Q Exercise Sport.

[43] Schmidt RA (1975). A schema theory of discrete motor skill learning.. Psychol Rev.

[44] Luppino G, Rizzolatti G (2000). The organization of the frontal motor cortex.. News Physiol Sci.

[45] Lawrence AD, Sahakian BJ, Robbins TW (1998). Cognitive functions and corticostriatal circuits: insights from Huntington’s disease.. Trends Cogn Sci.

[46] Gerardin E, Sirigu A, Lehéricy S, Poline JB, Gaymard B (2000). Partially overlapping neural networks for real and imagined hand movements.. Cereb Cortex.

[47] Jastorff J, Begliomini C, Fabbri-Destro M, Rizzolatti G, Orban GA (2010). Coding observed motor acts: different organizational principles in the parietal and premotor cortex of humans.. J Neurophysiol.

[48] Fernandez-Ruiz J, Goltz HC, DeSouza JFX, Vilis T, Crawford JD (2007). Human Parietal “Reach Region” Primarily Encodes Intrinsic Visual Direction, Not Extrinsic Movement Direction, in a Visual–Motor Dissociation Task.. Cereb Cortex.

[49] Battaglia Mayer A, Caminiti R (2002). Optic ataxia as a result of the breakdown of the global tuning fields of parietal neurones.. Brain.

[50] Parkinson A, Condon L, Jackson SR (2010). Parietal cortex coding of limb posture: In search of the body-schema.. Neuropsychologia.

[51] Haslinger B, Erhard P, Weilke F, Ceballos-Baumann AO, Bartenstein P (2002). The role of lateral premotor-cerebellar-parietal circuits in motor sequence control: a parametric fMRI study.. Cognitive Brain Res.

[52] Fleming MK, Stinear CM, Byblow WD (2010). Bilateral parietal cortex function during motor imagery.. Exp Brain Res.

[53] Cauda F, Giuliano G, Federico DA, Sergio D, Katiuscia S (2011). Discovering the somatotopic organization of the motor areas of the medial wall using low-frequency bold fluctuations.. Hum Brain Mapp.

[54] Solodkin A, Hlustik P, Chen EE, Small SL (2004). Fine modulation in network activation during motor execution and motor imagery.. Cereb Cortex.

[55] Seitz RJ, Roland PE, Bohm C, Greitz T (1990). Motor learning in man: a positron emission tomographic study.. Neuroreport: An International Journal for the Rapid Communication of Research in Neuroscience.

[56] Albouy G, Sterpenich V, Balteau E, Vandewalle G, Desseilles M (2008). Both the hippocampus and striatum are involved in consolidation of motor sequence memory.. Neuron.

[57] Soliveri P, Brown RG, Jahanshahi M, Caraceni T, Marsden CD (1997). Learning manual pursuit tracking skills in patients with Parkinson’s disease.. Brain.

[58] Li CR (2000). Impairment of motor imagery in putamen lesions in humans.. Neurosci Lett.

[59] Doya K (2000). Complementary roles of basal ganglia and cerebellum in learning and motor control.. Curr Opin Neurobiol.

[60] Prabhu G, Voss M, Brochier T, Cattaneo L, Haggard P (2007). Excitability of human motor cortex inputs prior to grasp.. The Journal of physiology.

[61] Haggard P (2008). Human volition: towards a neuroscience of will.. Nat Rev Neurosci.

[62] Ogawa K, Inui T (2011). Neural representation of observed actions in the parietal and premotor cortex.. Neuroimage.

