# Supplementary figures and images for: Parallel Alterations of Functional Connectivity during Execution and Imagination after Motor Imagery Learning
Source: PLoS One. 2012 May 18;7(5):e36052. doi: 10.1371/journal.pone.0036052 (PMC3356366; doi:10.1371/journal.pone.0036052)

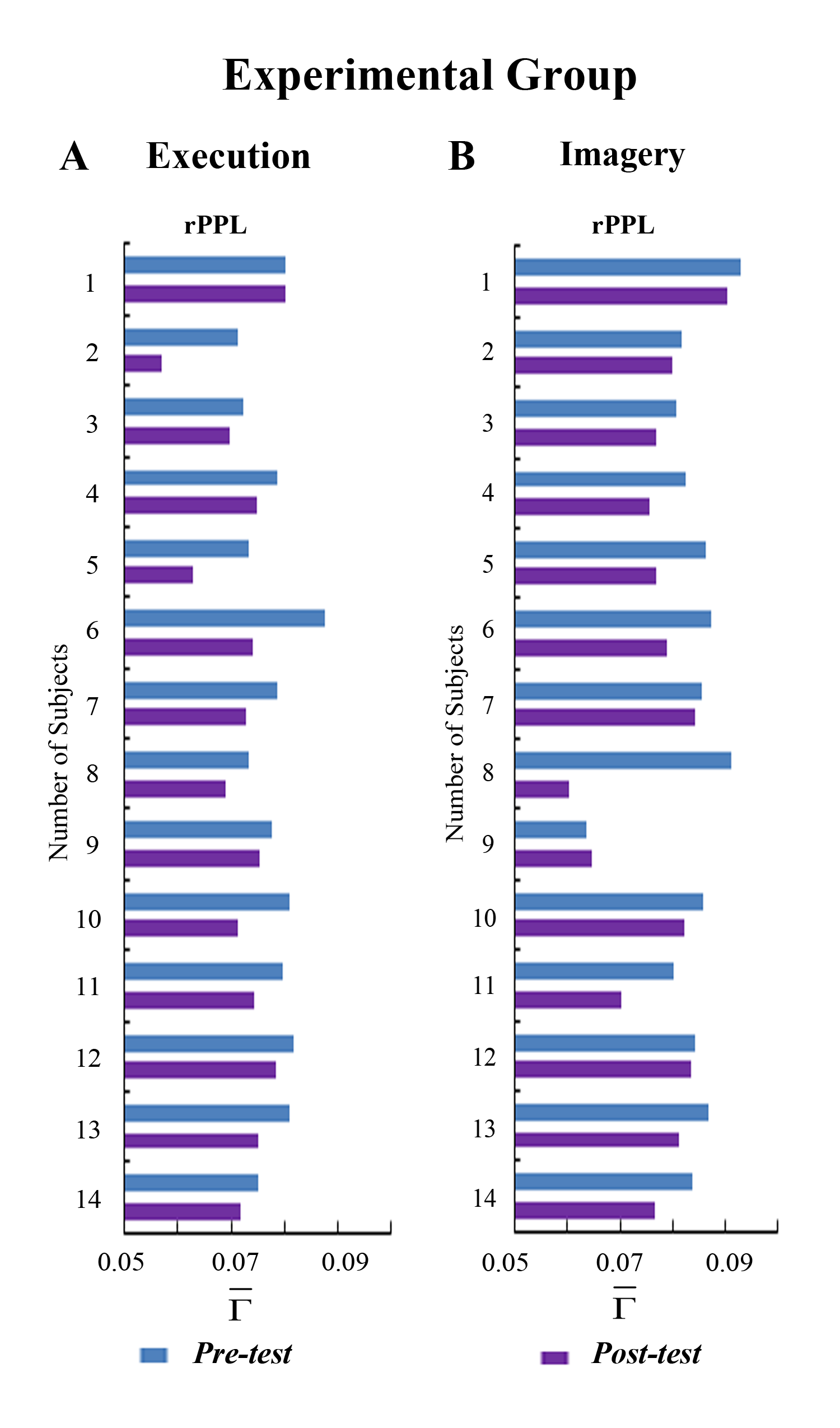

Supplement: Figure S1 — of the rPPL for each subject of the experimental group in motor execution/imagery task, (A) motor execution task, (B) motor imagery task. (TIF) [file pone.0036052.s001.tif]

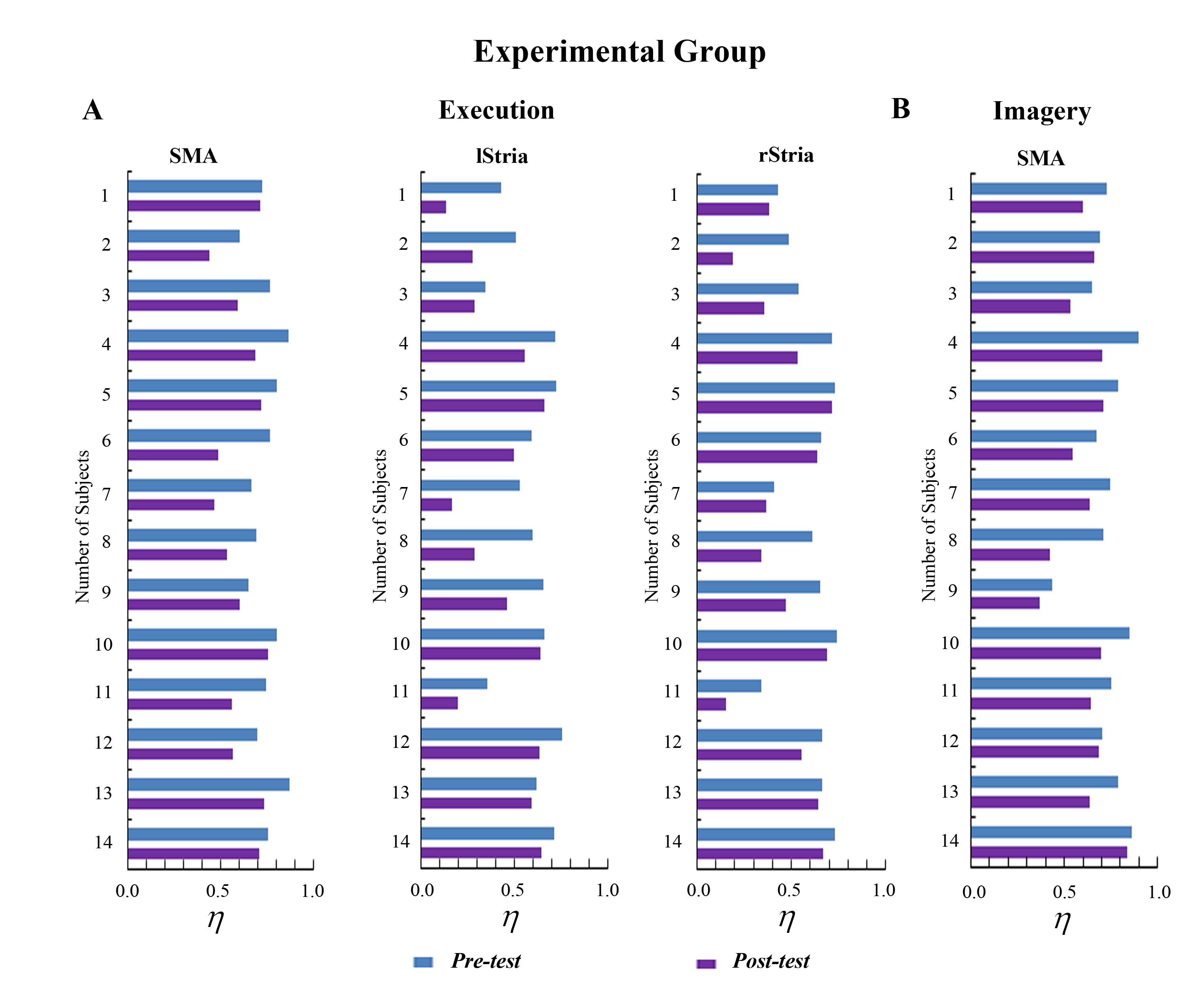

Supplement: Figure S2 — showing significant alteration between the rPPL and some other ROIs for each subject of the experimental group in motor execution/imagery tasks, (A) between the rPPL and SMA/left striatum/right striatum for each subject, motor execution task, (B) between the rPPL and SMA for each subject, motor imagery task. (TIF) [file pone.0036052.s002.tif]
