# Supplementary material for: Parallel Alterations of Functional Connectivity during Execution and Imagination after Motor Imagery Learning
Source: PLoS One. 2012 May 18;7(5):e36052. doi: 10.1371/journal.pone.0036052 (PMC3356366; doi:10.1371/journal.pone.0036052)
Supplement: Table S1 — The coordinates and t-value of the peak voxel within group ROIs for motor execution and motor imagery tasks at pre-test for the experimental group. (DOC) [file pone.0036052.s003.doc]

| **Region** | **L/R** | **BA** | **Pre-test** | | | | **Pre-test** | | | |
| --- | --- | --- | --- | --- | --- | --- | --- | --- | --- | --- |
|  |  |  | **Motor execution** | | | | **Motor imagery** | | | |
|  |  |  | x | y | z | t_max_ | x | y | z | t_max_ |
| PMA | L | 6 | -27 | -7 | 58 | 9.58 | -24 | -4 | 54 | 10.71 |
| PMA | R | 6 | 33 | -10 | 58 | 7.48 | 30 | -7 | 50 | 6.77 |
| M1 | L | 4 | -36 | -16 | 54 | 10.02 | -48 | -10 | 54 | 5.56 |
| M1 | R | 4 | 42 | -16 | 58 | 5.49 |  |  |  |  |
| PPL | L | 7 | -24 | -58 | 66 | 6.64 | -21 | -67 | 50 | 9.51 |
| PPL | R | 7 | 21 | -64 | 54 | 4.84 | 18 | -70 | 50 | 5.97 |
| SMA | L/R | 6 | -3 | -1 | 62 | 11.79 | -3 | 8 | 54 | 12.6 |
| Striatum | L |  | -24 | 2 | 2 | 8.53 | -24 | 2 | 2 | 8.57 |
| Striatum | R |  | 24 | 5 | 2 | 5.49 | 24 | 2 | 2 | 5.79 |
| Thalamus | L |  | -12 | -16 | 2 | 6.60 | -12 | -7 | 14 | 5.56 |
| Thalamus | R |  | 12 | -10 | 2 | 3.45 | 12 | -7 | 10 | 3.18 |
| Cerebellum | L |  | -27 | -58 | -30 | 7.24 | -33 | -61 | -30 | 5.91 |
| Cerebellum | R |  | 33 | -55 | -30 | 9.38 | 33 | -64 | -30 | 8.30 |

Note. MNI coordinates; Abbreviations: PMA—premotor area; M1—primary motor cortex; PPL—posterior parietal lobe; SMA—supplementary motor area; BA—Brodmann’s area.
