# Supplementary material for: Parallel Alterations of Functional Connectivity during Execution and Imagination after Motor Imagery Learning
Source: PLoS One. 2012 May 18;7(5):e36052. doi: 10.1371/journal.pone.0036052 (PMC3356366; doi:10.1371/journal.pone.0036052)
Supplement: Table S2 — The coordinates and t-value of the peak voxel within group ROIs for motor execution and motor imagery tasks at post-test for the experimental group. (DOC) [file pone.0036052.s004.doc]

| **Region** | **L/R** | **BA** | **Post-test** | | | | **Post-test** | | | |
| --- | --- | --- | --- | --- | --- | --- | --- | --- | --- | --- |
|  |  |  | **Motor execution** | | | | **Motor imagery** | | | |
|  |  |  | x | y | z | t_max_ | x | y | z | t_max_ |
| PMA | L | 6 | -27 | -7 | 58 | 7.02 | -39 | -13 | 54 | 8.78 |
| PMA | R | 6 | 36 | -13 | 58 | 6.26 | 48 | -4 | 58 | 6.85 |
| M1 | L | 4 | -36 | -16 | 54 | 9.89 | -39 | -16 | 58 | 6.93 |
| M1 | R | 4 | 39 | -16 | 58 | 6.47 |  |  |  |  |
| PPL | L | 7 | -27 | -58 | 58 | 5.67 | -27 | -58 | 58 | 4.98 |
| PPL | R | 7 | 24 | -64 | 50 | 3.60 | 24 | -61 | 50 | 1.91 |
| SMA | L/R | 6 | 0 | -1 | 62 | 9.49 | 0 | 2 | 62 | 10.21 |
| Striatum | L |  | -24 | -1 | -2 | 7.91 | -24 | 2 | 2 | 7.75 |
| Striatum | R |  | 21 | 2 | 2 | 5.29 | 24 | 2 | 2 | 5.27 |
| Thalamus | L |  | -15 | -10 | 14 | 7.04 | -9 | -16 | 2 | 5.00 |
| Thalamus | R |  | 15 | -10 | 14 | 4.76 | 12 | -7 | 6 | 4.16 |
| Cerebellum | L |  | -21 | -52 | -26 | 7.72 | -33 | -64 | -26 | 6.52 |
| Cerebellum | R |  | 33 | -55 | -30 | 8.75 | 33 | -58 | -30 | 7.09 |

Note. MNI coordinates; Abbreviations: PMA—premotor area; M1—primary motor cortex; PPL—posterior parietal lobe; SMA—supplementary motor area; BA—Brodmann’s area.
