# Supplementary material for: Parallel Alterations of Functional Connectivity during Execution and Imagination after Motor Imagery Learning
Source: PLoS One. 2012 May 18;7(5):e36052. doi: 10.1371/journal.pone.0036052 (PMC3356366; doi:10.1371/journal.pone.0036052)
Supplement: Table S3 — The coordinates and t-value of the peak voxel within group ROIs for motor execution and motor imagery tasks at pre-test for the control group. (DOC) [file pone.0036052.s005.doc]

| **Region** | **L/R** | **BA** | **Pre-test** | | | | **Pre-test** | | | |
| --- | --- | --- | --- | --- | --- | --- | --- | --- | --- | --- |
|  |  |  | **Motor execution** | | | | **Motor imagery** | | | |
|  |  |  | x | y | z | t_max_ | x | y | z | t_max_ |
| PMA | L | 6 | -33 | -13 | 58 | 13.84 | -24 | -4 | 54 | 12.28 |
| PMA | R | 6 | 39 | -10 | 58 | 10.65 | 30 | -7 | 50 | 9.27 |
| M1 | L | 4 | -33 | -16 | 58 | 11.29 | -33 | -16 | 54 | 5.15 |
| M1 | R | 4 | 39 | -16 | 58 | 5.02 |  |  |  |  |
| PPL | L | 7 | -24 | -58 | 66 | 9.29 | -21 | -67 | 50 | 7.99 |
| PPL | R | 7 | 27 | -58 | 58 | 5.68 | 18 | -70 | 50 | 4.75 |
| SMA | L/R | 6 | 0 | 2 | 58 | 12.11 | -3 | 8 | 54 | 9.32 |
| Striatum | L |  | -24 | 2 | 2 | 5.82 | -24 | 2 | 2 | 5.87 |
| Striatum | R |  | 24 | 5 | 2 | 3.34 | 24 | 2 | 2 | 4.28 |
| Thalamus | L |  | -12 | -16 | 2 | 7.41 | -18 | -10 | 6 | 5.07 |
| Thalamus | R |  | 12 | -10 | 2 | 3.76 | 15 | -7 | -2 | 4.39 |
| Cerebellum | L |  | -27 | -58 | -30 | 9.36 | -24 | -61 | -30 | 6.14 |
| Cerebellum | R |  | 33 | -55 | -30 | 10.73 | 27 | -61 | -30 | 7.54 |

Note. MNI coordinates; Abbreviations: PMA—premotor area; M1—primary motor cortex; PPL—posterior parietal lobe; SMA—supplementary motor area; BA—Brodmann’s area.
