# Supplementary material for: Parallel Alterations of Functional Connectivity during Execution and Imagination after Motor Imagery Learning
Source: PLoS One. 2012 May 18;7(5):e36052. doi: 10.1371/journal.pone.0036052 (PMC3356366; doi:10.1371/journal.pone.0036052)
Supplement: Table S4 — The coordinates and t-value of the peak voxel within group ROIs for motor execution and motor imagery tasks at post-test for the control group. (DOC) [file pone.0036052.s006.doc]

| **Region** | **L/R** | **BA** | **Post-test** | | | | **Post-test** | | | |
| --- | --- | --- | --- | --- | --- | --- | --- | --- | --- | --- |
|  |  |  | **Motor execution** | | | | **Motor imagery** | | | |
|  |  |  | x | y | z | t_max_ | x | y | z | t_max_ |
| PMA | L | 6 | -27 | -10 | 58 | 12.61 | -24 | -4 | 58 | 10.76 |
| PMA | R | 6 | 39 | -7 | 58 | 8.01 | 48 | -4 | 58 | 7.92 |
| M1 | L | 4 | -36 | -19 | 54 | 12.12 | -33 | -13 | 58 | 5.79 |
| M1 | R | 4 | 39 | -16 | 58 | 4.92 |  |  |  |  |
| PPL | L | 7 | -24 | -58 | 58 | 8.11 | -24 | -58 | 58 | 6.71 |
| PPL | R | 7 | 24 | -58 | 58 | 6.36 | 24 | -58 | 58 | 5.16 |
| SMA | L/R | 6 | 0 | 2 | 62 | 14.72 | 0 | 5 | 58 | 10.81 |
| Striatum | L |  | -30 | 8 | 2 | 4.69 | -30 | 8 | 2 | 6.78 |
| Striatum | R |  | 30 | 8 | 2 | 3.21 | 30 | 8 | 2 | 4.01 |
| Thalamus | L |  | -15 | -13 | 6 | 6.03 | -18 | -10 | 6 | 4.23 |
| Thalamus | R |  | 15 | -7 | 2 | 4.28 | 15 | -10 | 6 | 3.29 |
| Cerebellum | L |  | -21 | -58 | -26 | 9.64 | -24 | -64 | -30 | 4.72 |
| Cerebellum | R |  | 24 | -58 | -26 | 12.81 | 30 | -64 | -30 | 6.09 |

Note. MNI coordinates; Abbreviations: PMA—premotor area; M1—primary motor cortex; PPL—posterior parietal lobe; SMA—supplementary motor area; BA—Brodmann’s area.
