# Supplementary material for: Parallel Alterations of Functional Connectivity during Execution and Imagination after Motor Imagery Learning
Source: PLoS One. 2012 May 18;7(5):e36052. doi: 10.1371/journal.pone.0036052 (PMC3356366; doi:10.1371/journal.pone.0036052)
Supplement: Table S5 — The interaction effect between group and learning for each ROI in motor execution and motor imagery tasks. (DOC) [file pone.0036052.s007.doc]

| **Region** | **L/R** | **Motor execution** | | **Motor imagery** | |
| --- | --- | --- | --- | --- | --- |
|  |  | **F** | **P** | **F** | **P** |
| SMA | L/R | 0.227 | 0.638 | 0.055 | 0.817 |
| PPL | L | 0.771 | 0.389 | 0.044 | 0.836 |
| PPL | R | 6.480 | 0.018* | 5.574 | 0.027* |
| PMA | L | 4.009 | 0.057 | 0.681 | 0.417 |
| PMA | R | 0.578 | 0.455 | 1.822 | 0.190 |
| M1 | L | 0.640 | 0.431 | 0.153 | 0.700 |
| M1 | R | 1.450 | 0.240 |  |  |
| Striatum | L | 0.151 | 0.701 | 0.119 | 0.733 |
| Striatum | R | 2.067 | 0.163 | 0.300 | 0.589 |
| Thalamus | L | 0.616 | 0.440 | 0.000 | 0.986 |
| Thalamus | R | 0.014 | 0.906 | 0.165 | 0.688 |
| Cerebellum | L | 0.001 | 0.970 | 1.125 | 0.299 |
| Cerebellum | R | 0.122 | 0.730 | 0.479 | 0.496 |

Note. Abbreviations: SMA—supplementary motor area; PMA—premotor area; M1—primary motor cortex; PPL—posterior parietal lobe (* represents the significant alterations, p < 0.05).
